# Disseminated Nocardia farcinica Infection in a Renal Transplant Patient: A Case Report

**DOI:** 10.7759/cureus.54963

**Published:** 2024-02-26

**Authors:** Fatma Mustafa Alhashimi, Sara Salim, Asif Iqbal, Maida Balila, Muhammed Khalid Chishti

**Affiliations:** 1 College of Medicine, Mohammed Bin Rashid University of Medicine and Health Sciences, Dubai, ARE; 2 Department of Infectious Disease, Mediclinic City Hospital, Dubai, ARE; 3 Department of Neurosurgery, Mediclinic City Hospital, Dubai, ARE

**Keywords:** disseminated, transplant, immunocompromised, nocardiosis, nocardia

## Abstract

*Nocardia farcinica*, an aerobic, Gram-positive bacterium belonging to the genus *Nocardia*, is a challenging opportunistic pathogen, particularly impacting immunocompromised individuals. The prevalence of human disease has witnessed a notable rise over the past two decades, correlating with an expanding population of immunocompromised individuals and advancements in the detection and identification of *Nocardia* spp. within clinical laboratories. This case is of a 59-year-old male with compromised immunity due to immunosuppressive medication use following a renal transplant who had an array of presentations before confirming a diagnosis of disseminated nocardiosis. The challenges faced in our case provide valuable insights into the complexities associated with diagnosing and managing *Nocardia* infections in immunocompromised populations, informing future clinical practice and research endeavors.

## Introduction

Nocardiosis, caused by various species of the genus *Nocardia*, represents a unique challenge in the realm of infectious diseases. Although considered an opportunistic infection, its ability to mimic other common conditions often leads to diagnostic dilemmas, especially in immunocompromised individuals. This case report aims to shed light on a compelling instance of disseminated nocardial infection in a 59-year-old male with a history of immunosuppression.

Disseminated nocardiosis, an infrequently encountered yet potentially serious condition, is commonly linked to three widely recognized species: *Nocardia asteroides*, *N. brasiliensis*, and *N. farcinica *[1]. Nocardiosis constitutes a localized or widespread infection resulting from an aerobic actinomycete, primarily impacting individuals with compromised immune systems [2]. This condition manifests in patients undergoing immunosuppressive treatments, recipients of organ transplants, individuals with chronic pulmonary diseases, and those afflicted by human immunodeficiency virus (HIV) infection [3]. 

The prevalence of human disease has witnessed a notable rise over the past two decades, correlating with an expanding population of immunocompromised individuals and advancements in the detection and identification of *Nocardia* spp. within clinical laboratories [4]. Notably, instances of nocardiosis have emerged in acquired immunodeficiency syndrome (AIDS) patients, as well as in recipients of solid organ and bone marrow transplants [5]. Risk factors contributing to the susceptibility to nocardiosis in these patients encompass early graft rejection and intense immunosuppressive regimens [6]. Additionally, individuals afflicted with chronic granulomatous diseases and hematologic malignancies constitute a third category prone to an elevated risk of developing nocardiosis [4]. 

Our patient, a recipient of a cadaveric renal transplant due to end-stage renal disease, presented with a constellation of symptoms, including recurrent fever, right flank pain, and nausea. As an immunocompromised individual on a regimen of tacrolimus, mycophenolate, and prednisone, his clinical course and response to treatment pose intriguing challenges and lessons for the medical community.

In an era where immunosuppressive therapies are increasingly employed, the presented case contributes to the evolving landscape of infectious diseases, emphasizing the need for a comprehensive understanding of unusual pathogens such as *Nocardia*. This report aims to enhance medical awareness, prompt early recognition, and improve outcomes for patients facing the intricate interplay of immunosuppression and opportunistic infections.

## Case presentation

A 59-year-old male with compromised immunity sought emergency care due to the sudden onset of recurring fever, right flank pain, and nausea persisting for several days. Notably, he did not report any constitutional symptoms such as weight loss, chills, fatigue, or underlying diseases affecting various systems. His medical history included hypertension, type 2 diabetes mellitus, dyslipidemia, end-stage renal disease necessitating dialysis since July 2022, and autosomal dominant polycystic kidney disease, for which he underwent a cadaveric renal transplantation in December 2022. The patient was under immunosuppressive therapy comprising tacrolimus (5 mg twice daily), mycophenolate (1 g twice daily), and prednisone (5 mg daily). Other medications included nebivolol (5 mg daily), ezetimibe/atorvastatin (10 mg daily), trimetazidine (35 mg twice daily), and dapagliflozin/metformin (5 mg/1000 mg daily). He was stable regarding the primary graft function, had no postoperative complications, and was followed by the nephrology team.

On examination, the patient was noted to be febrile and tachycardic, and systemic examination was significant for right flank tenderness. Laboratory studies revealed the following: leukocytosis (WBC): 14.8 (4-11)×103/uL; neutrophilia: 13.07 (1.7-7.6)×103/uL; lymphopenia: 0.53 (1-3.2)×103/uL; elevated C-reactive protein (CRP): 186.8 (0-5) mg/L; elevated procalcitonin: 0.18 (low risk of sepsis >0.05 and <0.5) ng/mL; elevated creatinine: 146.1 (53-114.9) umol/L; low sodium (Na+): 127 (130-145) mmol/L; elevated potassium (K+): 5.2 (3.5-5.1) mmol/L; normal chloride (Cl-): 103 (98-107) mmol/L; and low bicarbonate (HCO3 -): 16 (22-29) mmol/L. Urine microscopic analysis revealed glycosuria as the only remarkable finding. Urine cultures revealed no growth. Blood cultures were taken and revealed no growth after five days. Although blood cultures showed no growth, a respiratory pathogen panel detected enterovirus. A chest X-ray detected an infiltrate in the lower lobe on the right side. Abdominal ultrasonography unveiled a sizable cystic lesion with multiple partitions, measuring 7x7x2.5 cm, positioned above the transplanted kidney, indicating a potential seroma. Further assessment through CT imaging of the abdomen and pelvis revealed polycystic kidneys displaying cysts with signs of hemorrhage or infection. Additionally, findings included a collection in the abdominal wall measuring 5x3 cm in the right iliac fossa and consolidation in the basal region of the right lung. Empiric antimicrobial therapy with levofloxacin 250 mg and piperacillin-tazobactam was initiated, leading to the clinical improvement of possible infectious-based collection and subsequent discharge.

**Figure 1:**
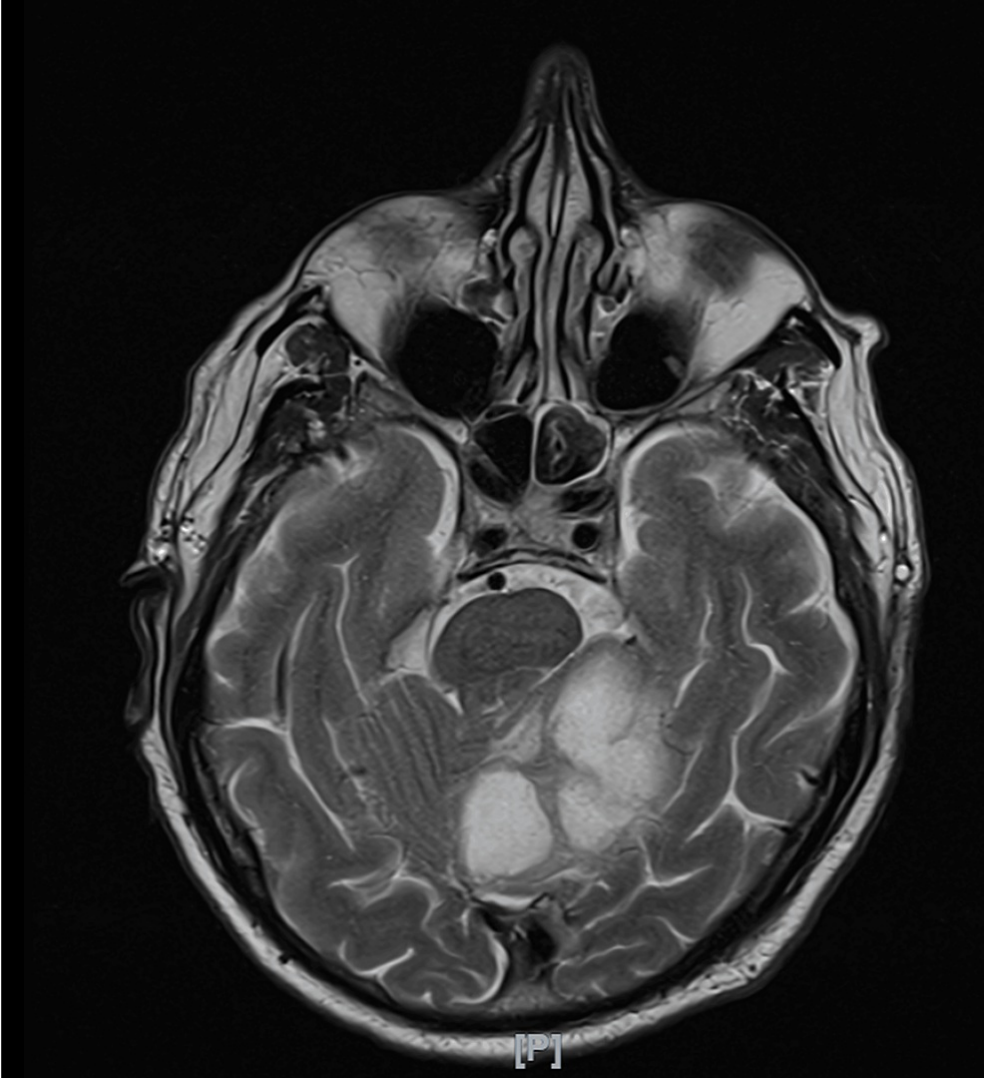
Results of the MRI of the brain in August 2023, revealing discernible lesions located in the cerebellum and left parietal lobe.

In August 2023, the patient returned with malaise, anorexia, nausea, and orthostasis, reporting a significant weight loss of 15 kg over the past two months. He also reported left-sided throbbing headaches with unsteady gait and loss of balance. Neurology consultation was sought, and amid concerns of venous sinus thrombosis, an MRI of the brain and MR venography (MRV) were performed, revealing lesions in the cerebellum and left parietal lobe (Figure 1).

Despite an unchanged right lower lobe infiltrate, further imaging revealed a round mass in the right lower lobe and multiple nodules in both lungs, raising suspicions of metastatic disease. Imaging also indicated intracranial lesions, prompting a scheduled CT-guided lung biopsy and a neurology consultation. Follow-up at the nephrology clinic revealed symptoms of an unsteady gait and loss of balance, necessitating further investigations, including a CT of the brain and an MRI of the spine. These tests revealed a large mass lesion in the left cerebellum and signs of metastatic disease in the spine. Given persistent symptoms of headache accompanied by nausea and vomiting, systemic glucocorticoid therapy was initiated. A PET-CT indicated cerebellar metastasis and lung nodules. 

Due to suspicion of possible lung primary and secondary brain metastasis, a decision was made to move forward with the CT-guided lung biopsy. However, a CT-guided lung biopsy confirmed benign pulmonary tissue, complicating the diagnostic picture. Further investigations, including brain biopsy, were recommended to exclude alternative causes of the lesions. MRI of the brain showed an increase in the size of the well-known lesion in the posterior fossa which now appeared multiloculated and ring-enhancing. The patient was admitted to the intensive care unit for a left posterior fossa burr hole and evacuation of the cerebellar lesion which was noted to be an abscess. Drainage of the abscess was done, and further investigation like Gram stain, culture including tuberculosis (TB), TB-PCR, and fungal culture was sent. Gram stain revealed gram-positive filamentous rods consistent with *Nocardia* species, prompting antimicrobial therapy with meropenem (1 g IV TID) and linezolid (600 mg IV BID) which was started following consultation with the infectious disease team as the patient has a documented allergy to trimethoprim-sulfamethoxazole. The patient experienced a mild to moderate allergic reaction to trimethoprim-sulfamethoxazole 15 years ago, resulting in a rash on the body and face. Subsequently, the patient has consistently declined to take the medication due to concerns about the potential reoccurrence of the allergic reaction. 

A follow-up MRI of the brain indicated no significant change in the appearance of the intraparenchymal lesion. Following the identification of *Nocardia farcinica* and its susceptibility profile, including susceptibility to amoxicillin-clavulanate, trimethoprim-sulfamethoxazole, linezolid, and amikacin, intermediate susceptibility to ceftriaxone, and resistance to imipenem and fluoroquinolone, a decision was made to undergo desensitization to trimethoprim-sulfamethoxazole. This procedure in consultation with an allergy specialist consultant, conducted in September 2023, proved successful, allowing for a transition in antimicrobial therapy to trimethoprim-sulfamethoxazole (15 mg/kg IV q8) and amoxicillin-clavulanate. The recommended course involves a minimum of three weeks of parenteral antimicrobial therapy, contingent upon clinical and radiographic responses to treatment, followed by combination oral therapy for at least one year, as advised by the infectious disease team. Considering the patient's immunocompromised state, lifelong secondary prophylaxis may be considered to prevent the recurrence of the infection.

**Figure 2:**
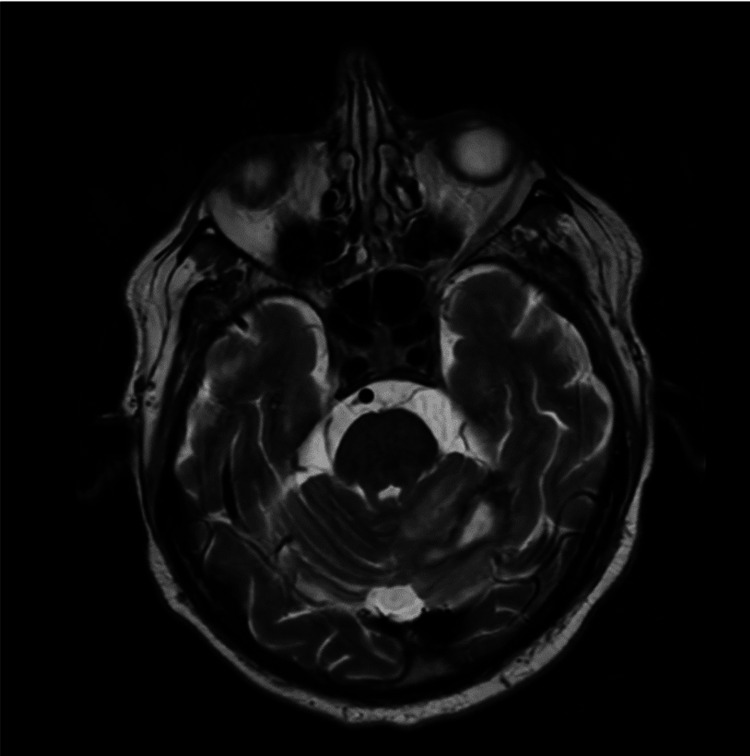
Outcomes of the MRI of the brain in October 2023, indicating a notable reduction in the size of the lesion situated in the left cerebellum.

Neurological symptoms resolved, and inflammatory markers decreased. Subsequent follow-up MRI of the brain with contrast in October 2023 demonstrated a reduction in the abscess size in the left cerebellum (Figure 2).

Upon achieving stability, the patient was discharged with a peripherally inserted central catheter (PICC) line for continued antibiotic therapy over six weeks, supported by home nurse assistance. Further follow-up with the infectious disease team is planned, considering the need for lifelong secondary prophylaxis. 

## Discussion

*Nocardia farcinica*, an aerobic, Gram-positive bacterium belonging to the genus *Nocardia*, is a challenging opportunistic pathogen, particularly impacting immunocompromised individuals [7]. The primary mode of acquiring pulmonary nocardiosis involves inhaling aerosolized organisms, while direct inoculation represents the second most common route, leading to cutaneous infections [8]. Individuals with compromised cell-mediated immunity face the highest risk of *Nocardia* infections, with additional risk factors encompassing systemic corticosteroid use, solid organ or hematopoietic stem cell transplantation, HIV infection, diabetes mellitus, and underlying malignancy treated with chemotherapy [9]. Extra-pulmonary dissemination commonly manifests in the brain, skin, and soft tissues, with less frequent involvement of bones, joints, or other organs [9]. This case report delves into the complex clinical landscape encompassing the diagnosis, therapeutic interventions, and clinical outcomes of disseminated *Nocardia farcinica* infection in the specific context of a renal transplant recipient.

The incidence of nocardiosis following solid organ transplantation is comparatively low, accounting for approximately 0.6% [9]. Specifically, the occurrence of *Nocardia* infections post-solid organ transplant ranges between 0.04% and 3.5%, with a more specific incidence in renal transplant patients ranging from 0.04% to 1.2% [10]. Noteworthy among the significant pathogenic strains are *N. asteroides*, *N. brasiliensis*, *N. otitidiscaviarum*, *N. farcinica*, *N. nova*, *N. cyriacigeorgica*, and *N. mexicana*. Notably, pulmonary nocardiosis is predominantly associated with *N. asteroides*, constituting approximately 85% of cases [11]. In contrast, *Nocardia farcinica* exhibits a preference for causing infections in the brain and skin [11].

Non-central nervous system (CNS) manifestations of nocardiosis commonly involve fever [6]. Cerebral nocardiosis is a rare opportunistic infection occurring predominantly in immunocompromised individuals, with intraparenchymal brain abscess formation being a characteristic outcome [12]. Mortality rates exceed 20% in immunocompetent patients and 55% in immunocompromised patients [13]. Diagnosis often relies on bacteriological confirmation through surgical excision of abscesses [4]. In disseminated cases, abscesses may form in various locations, with signs and symptoms tailored to the specific site, such as numbness or muscular weakness [6]. Additionally, signs of meningitis, including headache, stiff neck, or altered mental status, may be present in cases of CNS dissemination [6].

The clinical presentation in our renal transplant patient included nonspecific symptoms such as fever, malaise, and weight loss. Subsequently, the emergence of a headache later in the course of the disease posed an additional diagnostic challenge, underscoring the need for a high index of suspicion for opportunistic infections, especially in the post-transplant setting. Prompt recognition of the clinical deterioration and the performance of advanced microbiological studies was instrumental in identifying *N. farcinica* as the causative pathogen. Given the varied clinical manifestations of *Nocardia* infections, our case highlights the importance of thorough diagnostic evaluations, including imaging studies and microbiological testing, to guide appropriate treatment strategies.

Treatment initiation comprised a combination of trimethoprim-sulfamethoxazole and amoxicillin-clavulanate, determined by the antimicrobial susceptibility profile of the isolated strain. However, the commencement of this regimen was delayed upon the patient undergoing desensitization therapy owing to a documented allergy to trimethoprim-sulfamethoxazole. This necessity prolonged the time required to initiate the most appropriate antibiotic regimen. This comprehensive regimen, tailored to the specific susceptibility patterns, reflects the complexity of treating disseminated *Nocardia* infections. However, the optimal duration of therapy remains a topic of debate, and our case emphasizes the need for individualized treatment plans. Furthermore, it is imperative to consider potential drug interactions and adverse effects, particularly in the context of post-transplant immunosuppression. Close monitoring for medication tolerance and therapeutic drug monitoring play pivotal roles in preventing complications associated with long-term antimicrobial therapy.

Despite the initiation of appropriate treatment, disseminated *Nocardia farcinica* infections in immunocompromised individuals, such as renal transplant recipients, are associated with a guarded prognosis [14]. Our case highlights the importance of vigilant follow-up, encompassing clinical, radiological, and laboratory assessments, to detect any signs of relapse or complications. 

According to research findings, the one-year mortality rate following nocardiosis is approximately 25% [15]. Factors contributing to this mortality include the extent of infection involvement and the presence of comorbid conditions, both of which have been identified as significant associations with adverse outcomes. Therefore, long-term results in renal transplant patients with disseminated *Nocardia* infections remain variable, and a multidisciplinary approach involving infectious disease specialists, nephrologists, and transplant surgeons is essential for optimizing patient care.

## Conclusions

Our case report contributes to the growing evidence regarding disseminated *Nocardia farcinica* infections in renal transplant patients. It highlights the importance of early and accurate diagnosis, individualized treatment regimens based on susceptibility profiles, and attentive long-term follow-up. The challenges faced in our case provide valuable insights into the complexities associated with diagnosing and managing *Nocardia* infections in immunocompromised populations, informing future clinical practice and research endeavors.
